# Does socioeconomic status affect the association of social relationships and health? A moderator analysis

**DOI:** 10.1186/1475-9276-10-43

**Published:** 2011-10-13

**Authors:** Nico Vonneilich, Karl-Heinz Jöckel, Raimund Erbel, Jens Klein, Nico Dragano, Simone Weyers, Susanne Moebus, Johannes Siegrist, Olaf von dem Knesebeck

**Affiliations:** 1 Department of Medical Sociology and Health Economics, University Medical Center Hamburg-Eppendorf, Hamburg, Germany; 2 Institute of Medical Informatics, Biometry and Epidemiology, University Duisburg-Essen, Essen, Germany; 3 Clinic for Cardiology, University Clinic Essen, Essen, Germany; 4 Department of Medical Sociology, Heinrich Heine University Düsseldorf, Düsseldorf, Germany

## Abstract

**Background:**

Social relations have repeatedly been found to be an important determinant of health. However, it is unclear whether the association between social relations and health is consistent throughout different status groups. It is likely that health effects of social relations vary in different status groups, as stated in the hypothesis of differential vulnerability. In this analysis we explore whether socioeconomic status (SES) moderates the association between social relations and health.

**Methods:**

In the baseline examination of the Heinz Nixdorf Recall study, conducted in a dense populated Western German region (N = 4,814, response rate 56%), SES was measured by income and education. Social relations were classified by using both structural as well as functional measures. The Social Integration Index was used as a structural measure, whilst functional aspects were assessed by emotional and instrumental support. Health was indicated by self-rated health (1 item) and a short version of the CES-D scale measuring the frequency of depressive symptoms. Based on logistic regression models we calculated the relative excess risk due to interaction (RERI) which indicates existing moderator effects.

**Results:**

Our findings show highest odds ratios (ORs) for both poor self-rated health and more frequent depressive symptoms when respondents have a low SES as well as inappropriate social relations. For example, respondents with *low income and a low level of social integration *have an OR for a high depression score of 2.85 (95% CI 2.32-4.49), compared to an OR of 1.44 (95% CI 1.12-1.86) amongst those with a *low income but a high level of social integration *and an OR of 1.72 (95% CI 1.45-2.03) amongst respondents with *high income but a low level of social integration*. As reference group those reporting *high income and a high level of social integration *were used.

**Conclusions:**

The analyses indicate that the association of social relations and subjective health differs across SES groups as we find moderating effects of SES. However, results are inconsistent as nearly all RERI scores are positive but do not reach a significant level. Also moderating effects vary between women and men and depending on the indicators of SES and social relations used. Thus, the hypothesis of differential vulnerability can only partially be supported. In terms of practical implications, psychosocial and health interventions aiming towards the enhancement of social relations should especially consider the situation of the socially deprived.

## Background

So far, there has been extensive research revealing socioeconomic health inequalities across different societies [1-3]. Explanatory approaches of why such health inequalities do persist have, amongst others, focussed on social relations. Two reasons have been put forward to underline the argument: Firstly, social relations have been associated with socioeconomic status (SES) [4-6] and secondly, social relations have generally been recognised as an important social determinant of health [7-10].

Social relationships can affect health in different ways: Social ties might influence health-related behaviours, while social support might be valuable to cope with stressors [11]. Therefore, research on social relationships generally distinguishes between quantitative and qualitative aspects [9,12,13]. Quantitative characteristics of social relationships are, for instance, the frequency, intensity or permanence of social contacts. Measures of quantity are widely used in social-epidemiologic research and they usually form an index which provides information on the extent of social integration [14]. Moreover, concepts were developed and further promoted for assessing qualitative characteristics of social relationships. Social support is typically divided into subtypes, which include emotional and instrumental support [12]. Emotional support is related to understanding, esteem and help in decision-making, while instrumental support can be manifested in many forms, including practical help and financial support.

Numerous studies have shown an association of social relationships with morbidity as well as with self-rated health [9,12,15-18]. A recent meta-analytic review across 148 studies concludes that the influence of social relationships on mortality risks is comparable with well-established biomedical and behavioural risk factors [10]. Despite some inconsistencies it can be summarised that the effect of social relations on health can be found for the quantitative as well as the qualitative dimension of social relationships.

**Figure 1:**
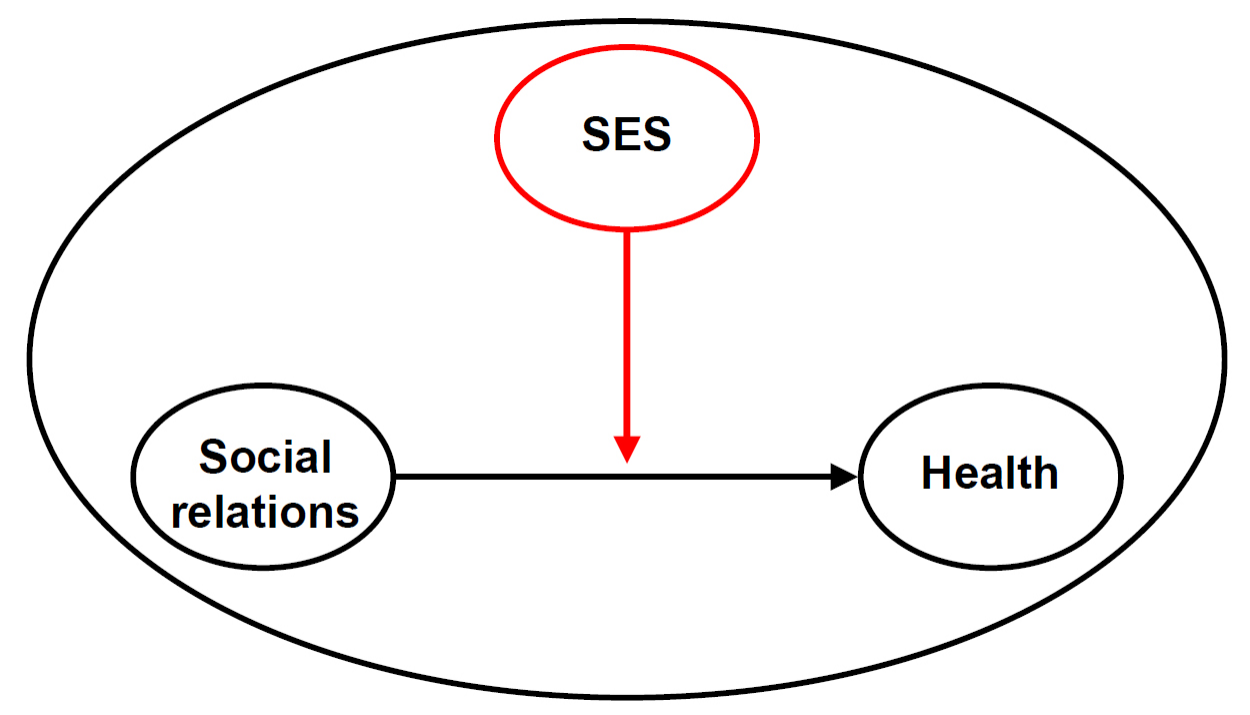
**The moderating effect of socioeconomic status (SES) on the association of social relations and health**.

Most of the studies analysing health effects of social relationships do not or only insufficiently consider socioeconomic factors. According to Krause [19], there are at least two ways in which socioeconomic variations in social relationships may be manifested: On the one hand it is possible that differences occur between socioeconomic status groups, regarding the extent of social relations; on the other hand it is likely that health-relevant effects of social relationships differ between status groups. In general, in the first mentioned case it is presumed that lower status groups have lesser or poorer social relationships (*differential exposure hypothesis*), for example less social contacts or less emotional support than higher SES groups. The latter case assumes higher vulnerability regarding adverse health effects of insufficient quantity and quality of social relationships in lower status groups (*differential vulnerability hypothesis*), i.e. social relations and health show stronger correlations in lower SES groups (see Figure 1). Results are inconsistent in terms of the differential vulnerability hypothesis [15,20].

With our analysis we try to shed light on the following research question: Does SES affect the association between social relationships and health? We assume that the link between social relations and health is affected by SES, i.e. that SES moderates the association of social relations and health. Functional as well as structural aspects of social relations are considered for the analysis as it was proposed for research in this area [13]. As there is evidence for gender differences in health inequalities as well as in health effects of social relations [16,20-25], it is likely that potential moderator effects differ between men and women.

## Methods

### Sample

Data stem from the baseline examination of the Heinz Nixdorf Recall (HNR) Study, which is an ongoing prospective population-based cohort study in an industrialised urban region (Ruhr Area) in Western Germany. Rationale, design and methods in this study have been described in detail elsewhere [26,27]. Respondents were recruited from the German population aged 45-75 years, living in three adjacent cities (Essen, Bochum and Mülheim/Ruhr). Recruitment was based on a random sample from mandatory citizen registries. Overall, 4,814 men and women participated in the study with a response rate of 56% [27]. Extensive baseline examinations were conducted from December 2000 to August 2003 and a five year follow-up has recently been completed. The main objective of the HNR study is to improve prediction of coronary heart disease by combining already established with new cardiovascular risk factors. SES, social relationships and health were assessed in the baseline screening as part of a social risk factor assessment by face-to-face interviews and paper-and-pencil questionnaires.

### Socioeconomic status (SES)

Income and education were used as SES measures. Education was classified according to the International Standard Classification of Education (ISCED) as total years of formal education, combining school and vocational training. This variable was then grouped into four categories, with 18 and more years of formal education as the highest category (equivalent to a university degree) and 10 or less years as the lowest category (equivalent to basic school education) [28].

Income was measured by equivalent household income including information on disposable income and size of household with number of adults and children according to OECD criteria, the so called 'OECD-modified scale' [29]. The respondent was attributed with a weight of 1, while every other member of the household was given a weight of 0.5.

For the analyses, both SES indicators were dichotomised (10 or less years of education versus more than 10 years of education, and low equivalent income of less than 1,000€ per month versus the higher income groups).

### Social Integration Index

The Social Integration Index (SII), which was originally constructed by Lisa F. Berkman [30], captures quantitative aspects of social relations. It includes the marital status respectively living with a partner, the number of contacts with close ties (including family members and friends) as well as the affiliation with voluntary associations. Each of these three domains score from 0 to 2 depending on the grade of integration: Marital status or cohabitation was scored 2, all else 0; number of close ties was scored 0 for 0-2 contacts, 1 for 3-11 contacts and 2 for 12 or more contacts; participation in voluntary associations was scored 0 for no participation, 1 for participation in one association, and 2 for participation in more than one voluntary association. The total score ranging from 0 to 6 was categorised into four levels of integration: Level I (Score 0-1), II (Score 2 and 3), III (Score 4 and 5) and IV (Score 6). These four levels of social integration were recoded into a dichotomised exposure variable with the levels I and II indicating low social integration versus high social integration (levels III and IV).

### Social Support

Measures of support include instrumental and emotional support. Both were assessed by a German adaptation of the New Haven EPESE questionnaire [31]. Instrumental support refers to help available in daily tasks, for example shopping, cooking, washing or others. Emotional support means having someone to talk to, someone to discuss problems with or someone who helps making difficult decisions.

First, questions of both support measures assessed the perceived availability of someone to help and the presence of one or more persons to approach when problems were experienced. In a second step respondents were asked who actually provided support and whether that support was appropriate. Based on the combination of information, four categories were built: 'Support not needed', 'support appropriate', 'support inappropriate' and 'support needed but not available'. Cut-points for the two measures of social support were chosen when either the support was inappropriate or when support was needed but not available, with all else representing appropriate social support.

### Health measures

Subjective health status and depressive symptoms were used as health indicators. Subjective health is a widely accepted measure for health, which has been linked to mortality and morbidity in a wide range of studies [32]. In the HNR study it was assessed by one question ('How would you, referring to the last twelve months, describe your overall health status?') on a 5-point Likert-scale ('very good', 'good', 'moderate', 'poor' and 'very poor'). Persons reporting moderate subjective health or worse were opposed to those with good subjective health or better.

Depressive symptoms were classified according to a short version (15 items) of the Centre for Epidemiological Study - Depression Scale (CES-D) [33,34]. This frequently applied screening instrument contains questions about the 7-day prevalence of different types of depressive symptoms (for example: 'During the past week I felt sad' or 'During the past week I felt anxious'). Answers are given on a 4-point Likert-scale ranging from 'less than one day' (0) to '5-7 days' (3). A total score of all items ranging from 0 to 45 was calculated, with higher values indicating a higher symptom load (Cronbach's alpha = 0.86 [35]). Depressive symptoms were dichotomised with gender specific and distribution-based cut-points, with a score in the upper-quartile representing a comparatively high frequency of depressive symptoms. Cut-points were 9 for men and 12 for women respectively [34].

### Statistical analysis

Logistic regression models were calculated with the dichotomised health measures subjective health and depressive symptoms as dependent variables. The two health measures were regressed on composite variables of the two dichotomised exposures of low social relations and low SES.

In order to detect a potential moderator effect of SES on the association between social relations and health, the relative excess risk due to interaction (RERI) score was calculated and is presented in the tables. Methodology and calculation of RERI is explained by Rothman and Greenland, who propose RERI as an approach for measuring interaction and therefore detect possible moderating effects in epidemiologic studies [36,37]. Interaction can be referred to as departure from additivity of effects on the chosen outcome scale, meaning the existence of super- or subadditivity of two different measures on a specific outcome. If there is no interaction, RERI equals 0. If there is superadditivity, RERI is > 0 and subadditivity will yield a RERI < 0. RERI is calculated using the following equation:

$$\text{RERI}=\text{OR}\left(\text{AB}\right)-\text{OR}\left(\text{A}{\text{B}}^{\prime }\right)-\text{OR}\left({\text{A}}^{\prime }\text{B}\right)+1.$$

In the equation, AB represents the negative health effect when both exposures, low SES and low social relations, persist, while AB' stands for the effect associated with low social relations only and A'B for the negative health effect of low SES. The RERI score is used here, as it is a clear and comprehensive measure of interaction effects. Moreover, the score facilitates the understanding of the direction of the interaction effect, a positive RERI score representing superadditivity and a negative score indicating subadditivity.

Hosmer and Lemeshow explained how to calculate confidence intervals in interaction analysis [37]. Analyses were conducted for the whole sample as well as for men and women separately. All statistical analyses were carried out using the PASW Statistics 18 program [38].

## Results

A description of the variables used is presented in Table 1. Results reveal that about a quarter of the population under study have a household equivalent income of less than 1,000€ per month and 11% report 10 years or less of formal education. Regarding the indicators of social relationships, women report being less socially integrated. Women also significantly more often lack appropriate instrumental support compared to men. General subjective health is distributed as follows: Altogether 47% rate their subjective health as good or better, while 53% find it moderate or worse. Men significantly more often report very good subjective health whilst women significantly more often show depressive symptoms.

**Table 1:** Distribution of variables

		Overall N (%)	Men N (%)	Women N (%)	p (Chi^2^)
**Total sample**		**4,814**	**2,395 (49.8)**	**2,419 (50.2)**	

***Variables ****(no. of missings)*					

**Age **(0)	Mean [SD]	59.6 [7.8]	59.8 [7.8]	59.5 [7.7]	

**Years of Education **(16)	< = 10 years	547 (11.4)	120 (5.0)	427 (17.7)	0.000

	11-13	2,676 (55.6)	1,139 (47.8)	1,537(63.6)	

	14-17	1,068 (22.2)	799 (33.5)	269 (11.1)	

	=> 18 years	507 (10.5)	325 (13.6)	182 (7.5)	

**Household equivalent income per month **(310)	< 1,000€	1,103 (24.5)	468 (20.4)	635 (28.7)	0.000

	1,000-1,500€	1,498 (33.3)	729 (31.8)	769 (34.8)	

	1,500-2,000€	1,027 (22.8)	572 (24.9)	455 (20.6)	

	> 2,000€	876 (19.4)	525 (22.9)	351 (7.8)	

**Social Integration Index **(110)	Level I (isolation)	375 (7.9)	112 (4.8)	263 (11.1)	0.000

	Level II	1968 (41.7)	919 (39.3)	1049 (44.1)	

	Level III	2,146 (45.5)	1,190 (50.9)	956 (40.2)	

	Level IV	229 (4.9)	119 (5.1)	110 (4.6)	

**Instrumental support **(69)	Not available/ inappropriate	602 (12.6)	252 (10.6)	350 (14.6)	0.000

	Not needed/ appropriate	4,163 (87.4)	2,115 (89.4)	2,048 (85.4)	

**Emotional support **(99)	Not available/ inappropriate	781 (16.4)	374 (15.8)	407 (17.0)	0.140

	Not needed/ appropriate	3,988 (83.6)	1,997 (84.2)	1,991 (83.0)	

**Subjective Health **(13)	Very good	372 (7.7)	213 (8.9)	159 (6.6)	0.000

	Good	1,898 (39.5)	995 (41.7)	903 (37.4)	

	Moderate	1,720 (35.8)	858 (36.0)	862 (35.7)	

	Poor	641 (13.4)	249 (10.4)	392 (16.2)	

	Very poor	170 (3.5)	70 (2.9)	100 (4.1)	

**Depressive Symptoms **(94)	Mean score [SD]	7.95 [6.11]	7.10 [5.42]	8.79 [6.62]	0.000

Table 2 presents the multivariate adjusted odds ratios (OR) for the overall sample: The three categories 'inappropriate social relations but high SES', 'appropriate social relations but low SES' and 'inappropriate social relations and low SES' are compared to the reference group 'appropriate social relations and high SES'.

**Table 2:** Multivariate adjusted^1 ^odds ratios of subjective health and depressive symptoms by social relations and socioeconomic status (SES, measured separately by income and education)

Overall sample	Subjective health (1 = moderate/poor)			Depressive symptoms (1 = Gender specific upper quartile)		
**Income**	***> = 1,000€***	***< 1,000€***	***RERI^2^***	***> = 1,000€***	***< 1,000€***	***RERI***

**Social** **Integration**						

High (Level III-IV)	1.00^a^	1.44 (1.16-1.77)	0.48 (-0.01-0.98)	1.00	1.44 (1.12-1.86)	**0.69^c^** **(0.32-1.06)**

Low (Level I-II)	1.41 (1.23-1.62)^b^	2.34 (1.94-2.82)		1.72 (1.45-2.03)	2.85 (2.32-3.49)	

**Instrumental** **Support**						

Not needed/appropriate	1.00	1.58 (1.36-1.83)	0.37 (-0.50-1.23)	1.00	1.63 (1.37-1.94)	0.54 (-0.88-1.95)

Inappropriate/not available	1.55 (1.25-1.93)	2.49 (1.81-3.43)		3.06 (2.43-3.84)	4.22 (3.11-5.73)	

**Emotional** **Support**						

Not needed/appropriate	1.00	1.49 (1.28-1.74)	1.04 (-0.03-2.11)	1.00	1.69 (1.41-2.03)	0.39 (-1.15-1.92)

Inappropriate/not available	1.81 (1.49-2.20)	3.35 (2.46-4.54)		3.95 (3.23-4.84)	5.03 (3.81-6.64)	

**Years of Education**	***> 10 years***	***=< 10 years***	***RERI***	***> 10 years***	***=< 10 years***	***RERI***

**Social** **Integration**						

High (Level III-IV)	1.00	1.74 (1.26-2.40)	0.11 (-0.66-0.87)	1.00	1.64 (1.13-2.37)	0.84 (-0.10-1.78)

Low (Level I-II)	1.40 (1.24-1.59)	2.25 (1.76-2.88)		1.74 (1.50-2.03)	3.22 (2.50-4.15)	

**Instrumental** **Support**						

Not needed/appropriate	1.00	1.71 (1.38-2.12)	0.25 (-1.01-1.50)	1.00	1.82 (1.44-2.30)	1.80 (-0.64-4.25)

Inappropriate/not available	1.63 (1.35-1.98)	2.59 (1.64-4.08)		3.12 (2.56-3.81)	5.75 (3.79-8.74)	

**Emotional** **Support**						

Not needed/appropriate	1.00	1.74 (1.40-2.17)	-0.17 (-1.32-0.98)	1.00	1.93 (1.51-2.46)	0.62 (-1.48-2.73)

Inappropriate/not available	2.03 (1.71-2.42)	2.60 (1.74-3.89)		3.91 (3.27-4.67)	5.46 (3.77-7.92)	

^1 ^adjusted for age and gender

^2 ^RERI: Relative excess due to interaction; RERI = OR(AB)-OR(AB')-OR(A'B)+1

^a ^Reference category

^b ^95% confidence interval

^c ^Bold RERI scores are significant on a p < 0.05 level

Highest odds ratios for reporting less than good subjective health and depressive symptoms can be found in nearly all cases for the 'inappropriate social relations and low SES'-group. Most RERI scores are positive, underlining the hypothesised superadditive interaction of the two exposures. Yet, only one score reaches statistical significance: A significant positive RERI score can be found for depressive symptoms in the group with low income and a low level of social integration.

The results regarding the two different indicators for SES show hardly any differences: ORs are very similar between income and education. Qualitative aspects of social relations i.e. support measures show strongest associations with the health measures used. While social integration - being a measure of quantitative aspects of social relationships - shows modest associations with both subjective health and depressive symptoms (ORs up to 1.72), associations between the measures of support and health indicators are rather strong (ORs up to 4.60).

Results in women are more consistent than they are in men (Tables 3 and 4). ORs for ill health are higher in women than in men when either social relationships are inappropriate or when both exposures, inappropriate social relations and low SES, persist. This pattern of stronger associations in women is emphasised by four significant and positive RERI scores. They indicate that the interaction of low income and inappropriate social relations is stronger than the addition of the two exposures.

**Table 3:** Multivariate adjusted^1^odds ratios of subjective health and depressive symptoms by social relations and socioeconomic status (SES, measured separately by income and education); results for men

Men	Subjective health (1 = moderate/poor)			Depressive symptoms (1 = Gender specific upper quartile)		
**Income**	***> = 1,000€***	***< 1,000€***	***RERI^2^***	***> = 1,000€***	***< 1,000€***	***RERI***

**Social** **Integration**						

High (Level III-IV)	1.00^a^	1.74 (1.30-2.34)	-0.17 (-0.89-0.56)	1.00	1.73 (1.23-2.44)	0.62 (-0.40-1.63)

Low (Level I-II)	1.35 (1.12-1.63)^b^	1.93 (1.45-2.56)		1.85 (1.48-2.33)	3.20 (2.36-4.35)	

**Instrumental** **Support**						

Not needed/appropriate	1.00	1.58 (1.26-1.97)	0.01 (-1.05-1.08)	1.00	1.78 (1.38-2.28)	0.12 (-1.71-1.95)

Inappropriate/not available	1.40 (1.01-1.93)	1.99 (1.24-3.18)		2.62 (1.86-3.70)	3.51 (2.20-5.62)	

**Emotional** **Support**						

Not needed/appropriate	1.00	1.56 (1.24-1.97)	0.04 (-1.03-1.11)	1.00	1.87 (1.43-2.43)	-0.23 (-2.13-1.68)

Inappropriate/not available	1.72 (1.31-2.26)	2.32 (1.54-3.50)		3.53 (2.65-4.70)	4.17 (2.78-6.26)	

**Years of Education**	***> 10 years***	***=< 10 years***	***RERI***	***> 10 years***	***=< 10 years***	***RERI***

**Social** **Integration**						

High (Level III-IV)	1.00	2.04 (1.04-3.99)	-0.48 (-2.10 -1.14)	1.00	3.85 (1.98-7.46)	-1.36 (-4.33-1.60)

Low (Level I-II)	1.32 (1.12-1.57)	1.88 (1.17-3.01)		1.89 (1.55-2.31)	3.37 (2.09-5.44)	

**Instrumental** **Support**						

Not needed/appropriate	1.00	2.04 (1.31-3.17)	**-1.46^c^** **(-2.79-** **-0.13)**	1.00	2.61 (1.69-4.05)	0.18 (-3.65-4.01)

Inappropriate/not available	1.53 (1.16-2.03)	1.11 (0.50-2.45)		2.68 (2.00-3.58)	4.47 (1.99-10.06)	

**Emotional** **Support**						

Not needed/appropriate	1.00	1.56 (1.00-2.42)	0.55 (-1.73-2.83)	1.00	2.59 (1.64-4.09)	2.01 (-3.33-7.36)

Inappropriate/not available	1.65 (1.30-2.09)	2.76 (1.26-6.04)		3.28 (2.56-4.19)	6.88 (3.22-14.70)	

^1 ^adjusted for age

^2 ^RERI: Relative excess due to interaction; RERI = OR(AB)-OR(AB')-OR(A'B)+1

^a ^Reference category

^b ^95% confidence interval

^c ^Bold RERI scores are significant on a p < 0.05 level

**Table 4:** Multivariate adjusted^1 ^odds ratios of subjective health and depressive symptoms by social relations and socioeconomic status (SES, measured separately by income and education); results for women

Women	Subjective health (1 = moderate/poor)			Depressive symptoms (1 = Gender specific upper quartile)		
**Income**	***> = 1,000€***	***< 1,000€***	***RERI^2^***	***> = 1,000€***	***< 1,000€***	***RERI***

**Social** **Integration**						

High (Level III-IV)	1.00^a^	1.15 (0.85-1.56)	**0.92^c^** **(0.27-1.57)**	1.00	1.18 (0.80-1.74)	**0.90** **(0.18-1.62)**

Low (Level I-II)	1.43 (1.17-1.74)^b^	2.49 (1.93-3.22)		1.60 (1.25-2.06)	2.68 (2.02-3.56)	

**Instrumental** **Support**						

Not needed/appropriate	1.00	1.51 (1.23-1.86)	0.73 (-0.61-2.07)	1.00	1.58 (1.24-2.02)	0.87 (-1.31-3.05)

Inappropriate/not available	1.62 (1.20-2.19)	2.87 (1.84-4.46)		3.57 (2.62-4.87)	5.02 (3.35-7.52)	

**Emotional** **Support**						

Not needed/appropriate	1.00	1.38 (1.12-1.70)	**2.67** **(0.29-5.06)**	1.00	1.63 (1.26-2.10)	0.96 (-1.50-3.42)

Inappropriate/not available	1.88 (1.42-2.49)	4.94 (3.05-7.99)		4.51 (3.37-6.02)	6.08 (4.14-8.95)	

**Years of Education**	***> 10 years***	***=< 10 years***	***RERI***	***> 10 years***	***=< 10 years***	***RERI***

**Social** **Integration**						

High (Level III-IV)	1.00	1.78 (1.22-2.60)	0.26 (-0.66-1.18)	1.00	1.11 (0.70-1.77)	**1.27** **(0.36-2.17)**

Low (Level I-II)	1.52 (1.27-1.82)	2.56 (1.90-3.45)		1.56 (1.25-1.95)	2.94 (2.15-4.01)	

**Instrumental** **Support**						

Not needed/appropriate	1.00	1.68 (1.31-2.16)	1.47 (-0.85-3.79)	1.00	1.60 (1.20-2.12)	2.03 (-1.04-5.12)

Inappropriate/not available	1.72 (1.32-2.24)	3.86 (2.14-6.97)		3.56 (2.71-4.68)	6.20 (3.80-10.12)	

**Emotional** **Support**						

Not needed/appropriate	1.00	1.87 (1.45-2.42)	-0.81 (-2.25 -0.63)	1.00	1.73 (1.29-2.32)	-0.51 (-2.91-1.89)

Inappropriate/not available	2.58 (1.98-3.37)	2.65 (1.66-4.23)		4.82 (3.72-6.25)	5.04 (3.27-7.77)	

^1 ^adjusted for age

^2 ^RERI: Relative excess due to interaction; RERI = OR(AB)-OR(AB')-OR(A'B)+1

^a ^Reference category

^b ^95% confidence interval

^c ^Bold RERI scores are significant on a p < 0.05 level

## Discussion

This is one of the first studies to analyse the effect of SES on the association of social relations and health (see [15,20,39,40]). Our results indicate that accumulation of the two exposures, low SES and inappropriate social relationships lead to highest health risks, measured by subjective health and depressive symptoms. The combination of low SES and inappropriate social relationships is related to a 2.25 to 5.75-fold increased odds ratio for ill health compared to persons reporting high SES and appropriate social relationships. In most cases the two exposures interact and their combined negative effect on health exceeds the addition of each exposure alone. Positive RERI scores point towards such moderating effects, even though most of the RERI scores remain insignificant. This ambiguity makes interpretation of results difficult: On the one hand a majority of the presented RERI scores are positive and highest odds ratios are found in the group with double exposure of inappropriate social relations and low SES. On the other hand most RERI scores remain insignificant. Therefore the hypothesis of differential vulnerability can only partially be supported.

Similarly ambiguous results have also been found in an earlier study with the same study population regarding the effects of social status on the association of social relations with health behaviour [41]. Only few studies have examined how socioeconomic factors might influence the association between social relations and health. In terms of potential moderating effects of SES on the association of social relations and health, a study by Knesebeck did not support the hypothesis of differential vulnerability [15], while Huure and colleagues found partial evidence [39]. Stronger evidence was found by Heritage and colleagues in a french study [40].

Concerning gender differences, evidence for a moderating effect of SES on social relations and health is particularly strong in women. Especially when SES is measured by income, significant moderating effects of income on the association between social relations and health are identified. A majority of the RERI scores are clearly positive and four RERI scores reach a significant level. Thus, low SES and inappropriate social relationships interact and their ORs for ill health potentiate rather than add up. These gender differences suggest that future studies on health inequalities and social relations should use gender-specific analyses.

Results vary depending on the indicators used. Qualitative aspects of social relations, i.e. emotional and instrumental support, show strongest associations especially with depressive symptoms. Mental health has been linked to social relationships before. Social isolation and loss of social ties have been found to be most potent predictors of depressive symptoms among the elderly [42]. Moreover, a similarity in measurement of depressive symptoms and social support might partly account for this association. If one feels rather depressed or alone, a tendency to deny availability of support is likely. Therefore, our results suggest that indicators of social relations and health should be chosen carefully as results differ depending on the indicators selected.

Overall, our results indicate that research on social relations and health and research on health inequalities should be brought together, as socioeconomic factors influence the association of social relations and health. Until now, most studies have either focused on the association of social relations and health or on socioeconomic inequalities in health, but these two strands have rarely been put together.

Translating our results into interventional practice, this would mean that interventions aiming at the reduction of health inequalities should also focus on social relations. Health benefits of interventions targeting on social relations would be more pronounced in low SES groups. Improving social relations in low SES groups could be one measure to reduce inequalities in health. Such improvements can be achieved on a structural as well as on an individual level. Structural aspects such as living conditions should include opportunities as for example public spaces, where people can get in contact with each other and which might help fostering social networks. On an individual level, measures to improve and promote social skills or sociability play an important role. While planning such interventions, one should keep in mind that structural aspects do shape our social environment. Therefore, focussing on the association of social relationships and health as possible target for intervention should not lead to neglect of social inequalities. An intervention on social relations may improve health of the socially deprived, but will not alter social inequalities which themselves influence population health.

The data used for our analyses has many merits. It is based on a large and unselected urban population of middle- and older-aged men and women. In the HNR study special emphasis was put on quality control of data collection and data handling, as evidenced by external certification [26]. Also, complex measures of social support indicators were used. When constructing the items for measuring support, both availability as well as adequacy of support were considered, as proposed in earlier research [43].

As the HNR Study specifically focused on the detection of arteriosclerosis as a risk factor of coronary heart disease (CHD), we additionally stratified our analyses for known CHD. Results indicate that the exclusion of persons with known CHD did not alter the results (results not shown here).

In terms of methodological limitations, potential graded effects between different status groups can not be displayed, as independent as well as moderator variable were dichotomised mainly for clarity of results. Also, the cross-sectional design does not allow conclusions concerning the temporal sequence of events. From a theoretical point of view it is considered that social relations affect health, though this causal direction can only be proven on the basis of a prospective study design. Compared to the population of the study area, there is a tendency towards an underrepresentation of lower status groups and a slightly higher proportion of persons with good health status [27], which in turn might lead to an underestimation of the associations of social relations and health. The nonsignificance of RERI-terms can partly be attributed to statistical limitations. RERI as a method of detecting interaction effects is robust, i.e. needs large differences in ORs, and depends on large sample sizes. Tests for nonadditivity as well as tests for other statistical interaction have very little power at typical sample sizes and their estimates lack precision [36].

## Conclusions

The results of the analysis presented show that research on the associations of social relations and health should consider socioeconomic factors, as these are likely to have impact on this association. The negative health effect of inappropriate social relationships is stronger among lower status groups compared to the effect in higher socioeconomic groups. Therefore, psychosocial and health interventions aiming towards the enhancement of social relations should consider the situation of the socially deprived.

## Competing interests

The authors declare that they have no competing interests.

## Authors' contributions

KHJ, RE, JS are principal investigators of the HNR study; OK is the principal investigator of the DFG-funded project 'Health inequalities and social relationships'. ND, SM and SW participated in conducting the HNR study. NV conducted the statistical analyses. NV, JK and OK were responsible for interpreting the data and writing the manuscript. All authors contributed to drafting the manuscript and approved the final version.
